# Supplementary material for: Modeling glioblastoma heterogeneity as a dynamic network of cell states
Source: Mol Syst Biol. 2021 Sep 16;17(9):e10105. doi: 10.15252/msb.202010105 (PMC8444284; doi:10.15252/msb.202010105)
Supplement: Supplementary file 5 — Source Data for Figure 3 [file MSB-17-e10105-s001.zip › Figure3A_sourcedata/GSEA_3065/hallmarks_state6.GseaPreranked.1623416308702/HALLMARK_ANGIOGENESIS.html]

Details for gene set HALLMARK\_ANGIOGENESIS[GSEA]

|  || Dataset | state6 |
| Phenotype | NoPhenotypeAvailable |
| Upregulated in class | na\_neg |
| GeneSet | HALLMARK\_ANGIOGENESIS |
| Enrichment Score (ES) | -0.7208384 |
| Normalized Enrichment Score (NES) | -2.0354824 |
| Nominal p-value | 0.0 |
| FDR q-value | 0.0 |
| FWER p-Value | 0.0 |
Table: GSEA Results Summary

  

Fig 1: Enrichment plot: HALLMARK\_ANGIOGENESIS      
 Profile of the Running ES Score & Positions of GeneSet Members on the Rank Ordered List

  

| PROBE | GENE SYMBOL | GENE\_TITLE | RANK IN GENE LIST | RANK METRIC SCORE | RUNNING ES | CORE ENRICHMENT || 1 | CCND2 |  |  | 32 | 0.269 | 0.0835 | No |
| 2 | STC1 |  |  | 1133 | 0.079 | -0.0162 | No |
| 3 | JAG1 |  |  | 2234 | 0.040 | -0.1285 | No |
| 4 | MSX1 |  |  | 4097 | -0.001 | -0.3402 | No |
| 5 | ITGAV |  |  | 4722 | -0.013 | -0.4071 | No |
| 6 | VAV2 |  |  | 5183 | -0.022 | -0.4524 | No |
| 7 | PDGFA |  |  | 5426 | -0.027 | -0.4713 | No |
| 8 | VEGFA |  |  | 5660 | -0.032 | -0.4875 | No |
| 9 | PTK2 |  |  | 6894 | -0.062 | -0.6079 | No |
| 10 | TNFRSF21 |  |  | 7096 | -0.068 | -0.6088 | No |
| 11 | FGFR1 |  |  | 8080 | -0.112 | -0.6844 | Yes |
| 12 | FSTL1 |  |  | 8373 | -0.143 | -0.6711 | Yes |
| 13 | LRPAP1 |  |  | 8498 | -0.161 | -0.6330 | Yes |
| 14 | COL5A2 |  |  | 8514 | -0.165 | -0.5813 | Yes |
| 15 | LPL |  |  | 8638 | -0.208 | -0.5279 | Yes |
| 16 | VCAN |  |  | 8664 | -0.219 | -0.4597 | Yes |
| 17 | TIMP1 |  |  | 8751 | -0.290 | -0.3755 | Yes |
| 18 | APP |  |  | 8758 | -0.300 | -0.2786 | Yes |
| 19 | SPP1 |  |  | 8779 | -0.338 | -0.1713 | Yes |
| 20 | POSTN |  |  | 8795 | -0.533 | 0.0001 | Yes |
Table: GSEA details [plain text format]

  

Fig 2: HALLMARK\_ANGIOGENESIS: Random ES distribution      
 Gene set null distribution of ES for **HALLMARK\_ANGIOGENESIS**
